# Lateral heterogeneity of soil physicochemical properties in riparian zones after agricultural abandonment

**DOI:** 10.1038/s41598-018-20723-4

**Published:** 2018-02-02

**Authors:** Huijuan Xia, Weijing Kong, Xuesen Li, Juntao Fan, Fen Guo, Osbert Jianxin Sun

**Affiliations:** 1 College of Forest Science, Beijing Forestry University, No. 35 Qinghua East Road, Haidian District, Beijing China; 2 Laboratory of Riverine Ecological Conservation and Technology, Chinese Research Academy of Environmental Sciences, No. 8 Dayangfang, Beiyuan Road, Chaoyang District, Beijing China; 3 State Key Laboratory of Environmental Criteria Risk Assessment, Chinese Research Academy of Environmental Sciences, No. 8 Dayangfang, Beiyuan Road, Chaoyang District, Beijing China; 4 Liaoning Province Linghe River Reserve Administration, No. 78 Zhujiang Road, Shuangta District, Shenyang City, Liaoning Province China

## Abstract

The study aimed to identify the lateral heterogeneity of soil physicochemical properties in riparian zones, and its underlying drivers during natural restoration after agricultural abandonment. Abandoned farmlands, after 5-year natural restoration, within 500 m from the edges on both sides of Liaohe River were selected as the study area. Soil physicochemical properties of four lateral buffers (<10 m, 10~100 m, 100~300 m, and >300 m from river edge, respectively) along riparian zones were measured. The results showed that riparian soils were characterized by high sand content (78.88%~96.52%) and poor soil nutrients. Soil silt content, organic carbon (OC), cation exchange capacity (CEC), total nitrogen (TN), and available nitrogen (AN) increased laterally with increasing distance from river edge, while soil sand content decreased. Total phosphorus (TP) and available phosphorus (AP) are not spatially autocorrelated. Soil OC, TN, AN, and CEC along upstream and midstream reaches showed negative spatial autocorrelation along the lateral gradients, and positive along downstream reach. Altitude, distance from river edge and distance from nearest farmland were the pronounced factors affecting soil physicochemical properties in this study.

## Introduction

Understanding the ecosystem succession of abandoned farmlands is important for the conservation of biodiversity, mitigation of soil erosion, carbon sequestration, and water retention^1^. Following agricultural abandonment, in general, vegetation could be restored through natural restoration (secondary succession) or active restoration (seed sowing or tree plantations)^2,3^. Secondary succession has attracted increasing attention for its higher natural value and lower cost^4,5^. To date, most researches on agricultural abandonment focused on the temporal dynamics of soil organic carbon, nitrogen, phosphorus^6–8^, vegetation composition and plant species diversity^9,10^. There is evidence that secondary succession after agricultural abandonment leads to significant changes in soil physical and chemical properties^3^. As the substratum for organisms living in, soils affect the distribution and diversity of plant species and even the performance of individual organisms^11^.

Soils are spatially heterogeneous as a consequence of combined actions of physical, chemical, or biological processes that act at different scales^12–14^. The spatial heterogeneity of soil properties is mainly affected by parent material, topography, vegetation, climate, biological conditions, and human activities such as land use changes and agriculture^15^. Previous studies mainly focused on the response of soil heterogeneity to land use and topography in terrestrial ecosystems^16–19^. However, the spatial heterogeneity of soil properties in riparian zones, the line-shaped system, was likely related to microtopography, vegetation, and the directional effect of environmental gradients related to flood^11^. For example, soil organic carbon and nitrogen in natural riparian zones typically increased with distance from river^14,20,21^. In contrast, soil total carbon and nitrogen in riparian zones during active restoration decreased with distance from river^22^. However, the lateral heterogeneity of soil properties in riparian zones during natural restoration after agricultural abandonment is poorly understood.

Generally, the geographic variables are spatially dependent (spatial autocorrelation)^23^, and the spatial heterogeneity of variables results from both extrinsic and intrinsic factors^24^. Spatial autocorrelation has been used to express the degree of dependencies among neighboring observations and the spatial heterogeneity of soil properties in farmlands^25^, grasslands^26^, alluvial floodplain^27^, and wetlands^14^. Whether riparian zones during natural restoration follows the same pattern as the above ecosystems is not clear to our best knowledge. Therefore, Liaohe River Reserve, established in 2010, was selected as study area to study the spatial heterogeneity in riparian soils. Farmlands within 500 m from the edges on both sides of Liaohe River were abandoned in 2010 and restored through natural restoration. Our study was conducted in riparian zones that exposed to agricultural abandonment, and the main objectives were to answer the following questions: (1) how soil physicochemical properties change along lateral gradients in riparian zones during natural restoration? (2) What are the determinants affecting the lateral heterogeneity of riparian soil physicochemical properties?

## Materials and Methods

### Study area

**Figure 1:**
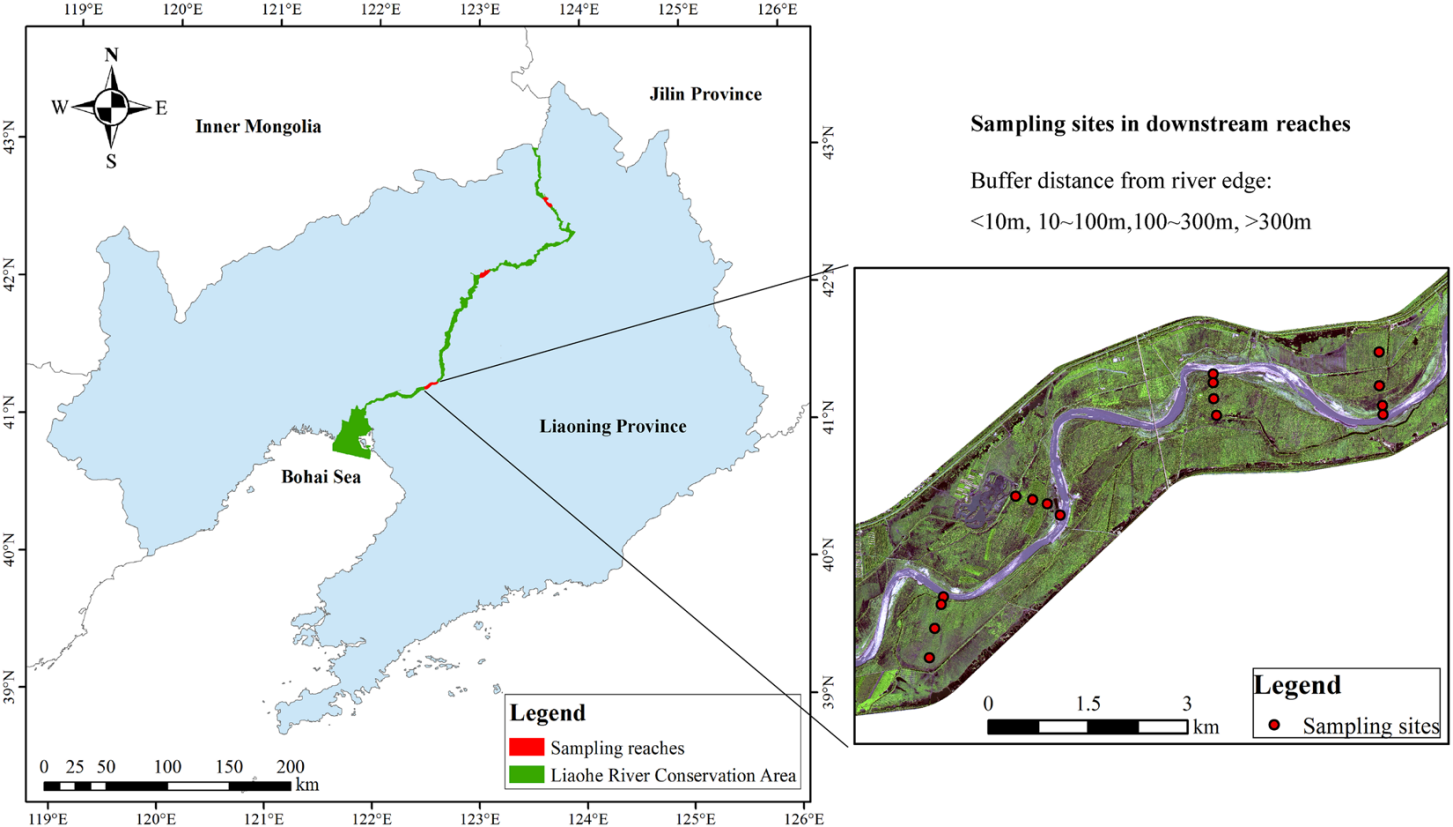
Sketch map of Liaohe River Reserve and sampling sites along downstream reach. Three reaches distributed in upstream, midstream and downstream were selected, respectively (as shown in the left map). The locations of sampling sites along downstream reach were shown as an example in the right map. Four sampling transects were established along each river reach, and four sample sites (<10 m, 10~100 m, 100~300 m, and >300 m from river edge, respectively) were established at each transect. The base map (right) is products of Wordview II (WV2) provided by Digitalglobe (https://discover.digitalglobe.com). ArcGIS 10.1 software (http://www.esri.com/software/arcgis) was used to develop the map by the first author (H.X.).

Liaohe River reserve is located in Liaoning Province, between 123°55.5′–121°41′E, 43°02′–40°47′N (Fig. 1). It originates from the confluence of East and West Liaohe River, flowing about 538 km to the Inlet of Bohai Sea in Panjin city, and covers an area of 1869.2 km^2^. Before 2010 riparian area of the river was covered by farmland. Established at 2010, the reserve was recovered mainly by natural recovery inside 500 m away from the river course on both sides, and the 500 m natural recovery area was fenced avoiding anthropogenic disturbance. The climate is warm-temperate with semi-humid continental monsoon climate. The warmest month is July with the temperature ranging from 20 to 30 °C, whereas the coldest month is January with the temperature ranging from −18 to −10 °C. Mean annual precipitation ranges from 400 to 1,000 mm, 75% of which occurs between June and September. The mean annual evaporation increases from southeast to northwest, ranging from 110 to 250 mm. The topography is predominately flat with a single landform, namely, alluvial plain.

The study was conducted in three reaches of upstream, midstream and downstream in Liaohe River reserve (Fig. 1). Predominant land-use along upstream and midstream reaches is farmland, which is located beyond 500 m from the river edges on both sides. Within 500 m from the edges on both sides (fenced area) of river, grassland is predominant. Riparian vegetation in fenced area along upstream reach is dominated by *Setaria glauca* and *Artemisia annua*, and *Calamagrostis epigeios*, *Artemisia lavandulaefolia* and *Artemisia annua* along midstream reach. Riparian zones along downstream reach are occupied by grassland either within or beyond fenced area, and *Phragmites australias* and *Artemisia annua* are the dominant species. Riparian vegetation along upstream reach is 5 to 10 cm high and 50% to 70% coverage, 30 to 80 cm high and 75% to 90% coverage along middle reach, and 100 to 170 cm high and 100% coverage along downstream reach.

### Soil and vegetation sampling

Liaohe River Reserve is mainly made up of two land-use types: one farmland, the other abandoned farmland. Soil samples were collected from abandoned farmlands along three reaches located in upstream, midstream and downstream, respectively (Fig. 1). Four transects separated by ~2 km were established perpendicular to the direction of water flow along each reach, and 12 transects in total. Riparian plants in Liaohe River Reserve changed laterally from annual mesic sedges and grasses to perennial xeric species. At each transect, lateral variation of plant life forms allowed us to establish sampling sites with a range of buffer distances from river edge: <10 m, 10~100 m, 100~300 m, and >300 m (Fig. 1). In total, 48 sites were sampled in September 2015, and all sites were exposed to agricultural abandonment based on the land-use change after the establishment of Liaohe River Reserve. At each sampling site, three soil cores were collected from the 0–15 cm soil layer, and soil cores were combined and mixed to get a composite soil sample. About 30 g from each composite soil sample were put into a pre-weighted aluminum box in order to determine soil moisture in the laboratory. The rest soil samples were placed in ventilated bags, and brought back to the laboratory. The dominant species and main companion species of plant communities at each sampling site were recorded. Plants were identified to species level according to *Flora of China*^28^, and unknown species were collected and identified in the laboratory.

### Soil physicochemical analysis

Soil samples were air-dried, then ground and passed through 1 mm and 0.149 mm sieves, respectively. Soil physical properties, including soil moisture and soil texture, and chemical properties, including pH, conductivity, OC, CEC, TN, AN, TP, and AP were measured.

Soil moisture was measured by oven drying aluminum boxes with fresh soil at 105 °C to a constant weight^29^. Soil texture was measured using Malvern laser particle size analyzer (Mastersizer-2000). Soil pH and conductivity were measured in soil extracts (10 g soil: 50 mL H_2_O) after vigorous agitation for 1~2 minutes and a settling period of 30 minutes^29^, using Horiba portable multi-parameter water quality analyzer (D-74). OC was measured by low-temperature external-heat K_2_Cr_2_O_7_ oxidation–photo-colorimetric method^29^. CEC was measured by BaCl_2_-H_2_SO_4_ method^30^. TN and TP were measured by alkaline K_2_S_2_O_8_–ultraviolet spectrophotometric method^31^. AN was measured using alkaline hydrolysis diffusion method^29^. AP was measured by NaHCO_3_ method^29^.

### Environmental data collection

The longitude, latitude, and altitude of each sampling site were measured with global positioning system (GPS). The distance of sampling sites from nearest residence (D-residence), distance from nearest farmland (D-farmland), and distance from river edge (D-river) were measured with Euclidean distance tool in ArcGIS 10.1. D-residence and D-farmland were the indicators of human disturbance, and D-river was the indicator of hydrologic disturbance.

### Statistical analysis

Means and standard errors for each soil physicochemical property were calculated in each sampling buffer. The means in each reach were compared using one-way ANOVA to test the significant differences of soil physicochemical properties between the four sampling buffers (<10 m, 10~100 m, 100~300 m, and >300 m). Pearson’s correlation analysis was employed to assess the relationships between environmental factors and soil physicochemical properties. All the above analysis was conducted in SPSS statistics 17.0 and the results were plotted using SigmaPlot 10.0.

In order to identify the main environmental factors that affected soil physicochemical properties, ordination analysis was conducted using CANOCO 4.5 software. First, detrended canonical analysis (DCA) was used to check the length of each axis^32^. If the length of longest axis was larger than 4.0, unimodal methods were more appropriate. On the other hand, if the longest gradient was shorter than 3.0, the linear methods were selected. And if the longest axis length was between 3.0 and 4.0, both types of methods were reasonable^32^. Since the longest axis in the study was 0.319, a constrained linear method redundancy analysis (RDA) was chosen for our data. All data were log-transformed and centered by species. The Monte Carlo test with 499 permutations was run to determine the significance of the RDA model.

Moran Index (*I*), as the conventional index^33^, was selected to evaluate the spatial autocorrelation that existed among soil samples taken from each reach. *I* range from −1 to 1. *I* greater than 0 indicate a positive autocorrelation, and otherwise *I* less than 0 indicate a negative autocorrelation. *I* near zero indicate samples with little discernible pattern in the spatial arrangement of values, or spatial randomness^34^. Moran’s *I* analysis was conducted in GS+ 5.0 software.

## Results

### Vegetation and soil properties

Nine plant communities were recorded in all sampling sites. Plant communities close to the river edge were dominated by annual plants, such as sedges and grasses that were short and hygrophytic. As the distance of sampling sites from river edge increased, mesic communities, i.e., *Artemisia annua*, *Calamagrostis epigeios*, and *Setaria glauca* appeared, followed by tall Compositae community, such as perennial *Cirsium setosum* and invasive species *Ambrosia trifida*.

Riparian soils in Liaohe River Reserve had high sand content (78.88%~96.52%) and low silt and clay contents. Soil moisture varied from 1.20% to 29.02%. The pH values indicated the neutral and slightly alkaline soil conditions in Liaohe River Reserve. Soil conductivity ranged from 1.45 ~ 11.03 ms.m^−1^. Five years after natural restoration, riparian soils had low nutrient contents. According to the classification criterion of soil nutrients in China, the mean concentrations of soil OC, TN, TP, AN, and AP were all at 5th or lower nutrient level (OC < 10 mg.g^−1^, TN < 500 mg.kg^−1^, TP < 200 mg.kg^−1^, AN < 30 mg.kg^−1^, AP < 3 mg.kg^−1^), indicating Liaohe River Reserve suffer from serious lack of soil nutrients.

### Lateral heterogeneity of riparian soils

**Figure 2:**
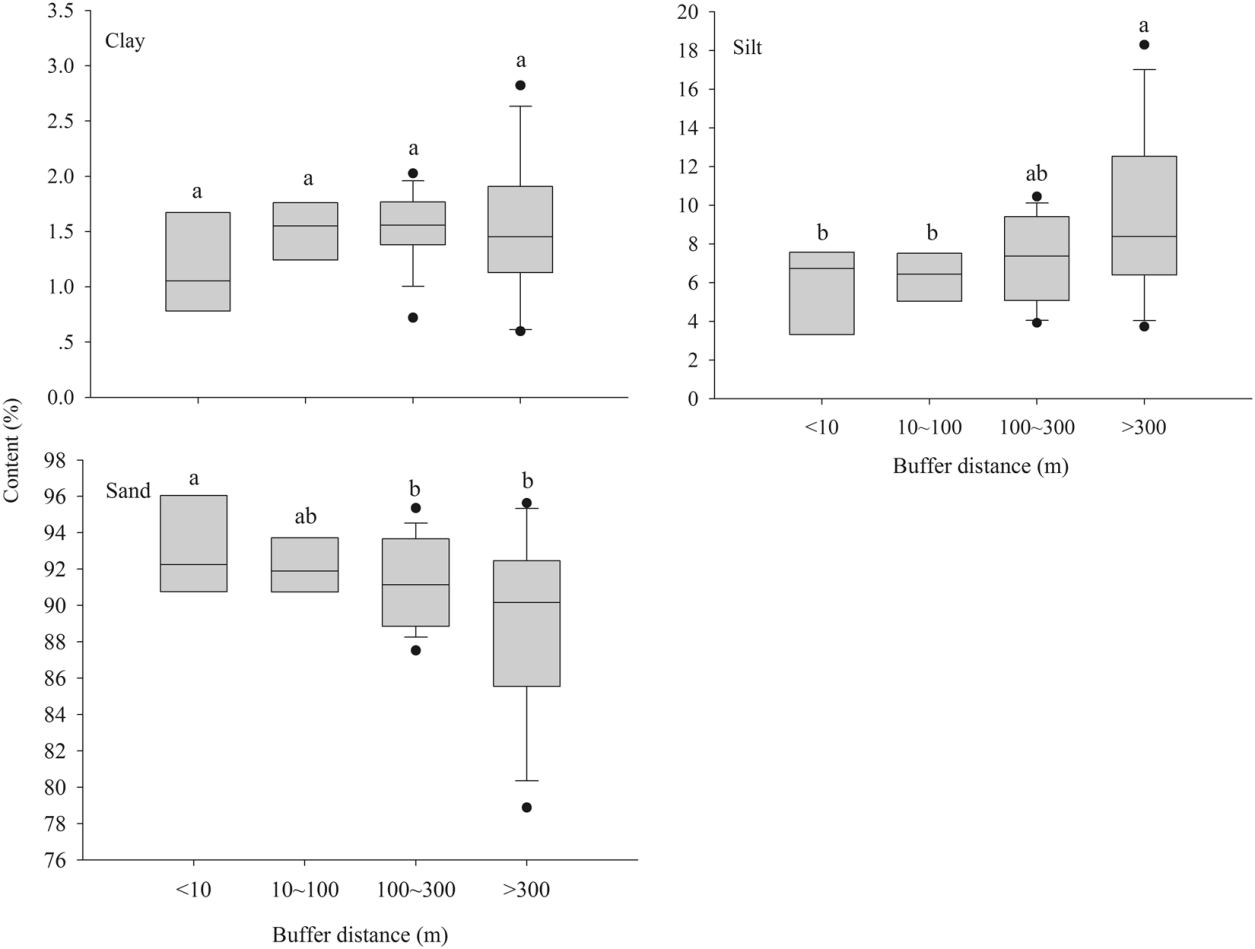
Box-plots of soil texture (Sand, Silt and Clay). The box-plots depict the median (horizontal line in each box), the upper and lower quartiles (box), and outliers (circles) of the data for soil texture. Lowercase letters above each bar indicate significant differences at the 0.05 level.

**Figure 3:**
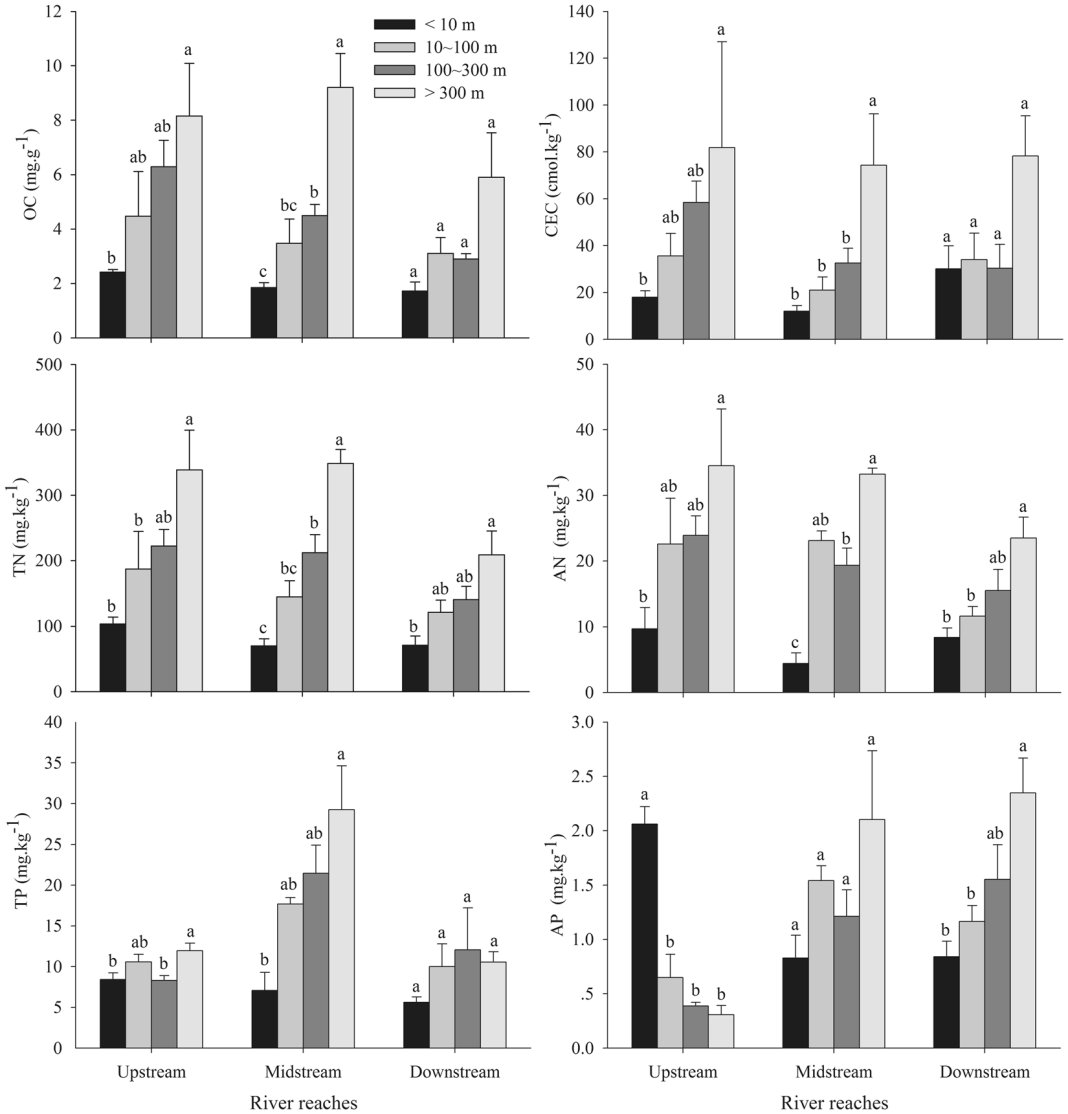
Lateral heterogeneity of soil nutrient contents. Values are means ± SE of 4 sampling sites in each buffer. Lowercase letters above each bar indicate significant differences in each river reach at the 0.05 level. OC: organic carbon, CEC: cation exchange capacity, TN: total nitrogen, AN: available nitrogen, TP: total phosphorus, AP: available phosphorus.

Silt content increased significantly with increasing buffer distance from river edge, and sand content showed the opposite trend (*p* < 0.05; Fig. 2). Clay content, soil moisture, pH, and conductivity showed no significant difference among four buffers (*p* > 0.05). Soil OC, CEC, TN, and AN contents showed increasing trends with distance from river edge (Fig. 3). Soil OC, CEC, and TP along downstream reach, and soil AP along midstream reach showed no significant lateral heterogeneity (*p* > 0.05). Soil AP close to river edge was significantly higher than that in other three buffers along upstream reach (*p* < 0.05).

### Relationships between soil properties and environmental factors

Soil silt and sand contents had significant correlations with D-river and D-residence (*p* < 0.01; Table 1), while soil clay content and moisture had no significant correlations with all environmental factors (*p* > 0.05). Clay content was positively correlated with OC, TN, TP, and AN (*p* < 0.05). Silt content was positively correlated with OC, TN, TP, AN, and CEC (*p* < 0.05), while sand content was negatively correlated with them (*p* < 0.05). Soil moisture was positively correlated with TP (*p* < 0.01).

**Table 1:** Correlations between soil properties and environmental factors.

Soil properties		Environmental factors				Soil physical properties			
		Altitude	D-river	D-farmland	D-residence	Clay	Silt	Sand	Moisture
Chemical properties	pH	−0.02	−0.08	0.28**	0.12	−0.24	−0.27	0.28	−0.26
	Conductivity	0.36**	−0.06	−0.24*	−0.22*	0.10	0.20	−0.19	−0.13
	OC	0.24*	0.58**	−0.45**	−0.25*	0.31*	0.56**	−0.54**	0.04
	TN	0.20	0.33**	−0.42**	−0.35**	0.32*	0.54**	−0.52**	0.17
	TP	0.07	−0.01	−0.35**	−0.45**	0.29*	0.35*	−0.35*	0.37**
	AN	0.19	0.35**	−0.43**	−0.36**	0.38**	0.58**	−0.57**	0.12
	AP	−0.13	0.25*	−0.09	−0.25*	−0.25	0.02	0.02	−0.21
	CEC	0.04	0.61**	−0.19	−0.07	0.08	0.33*	−0.30*	−0.09
Physical properties	Clay	0.02	0.11	−0.16	−0.25				
	Silt	0.00	0.41**	−0.28	−0.45**				
	Sand	0.00	−0.37**	0.27	0.44**				
	Moisture	0.12	0.15	−0.02	−0.14				

OC: organic carbon, TN: total nitrogen, TP: total phosphorus, AN: available nitrogen, AP: available phosphorus, CEC: cation exchange capacity, D-river: distance of sampling sites from river edge, D-farmland: distance from nearest farmland, D-residence: distance from nearest residence. **p < 0.01; *p < 0.05.

The correlation analysis between soil chemical properties and environmental factors indicated that, altitude was positively correlated with conductivity and OC (*p* < 0.05; Table 1). D-river was positively correlated with OC, TN, AN, AP, and CEC (*p* < 0.05). D-farmland exhibited significant correlations with pH, conductivity, OC, TN, TP, and AN (*p* < 0.05). D-residence did not show significant correlations with soil pH and CEC (*p* > 0.05), but was negatively correlated with the other chemical properties (*p* < 0.05).

**Figure 4:**
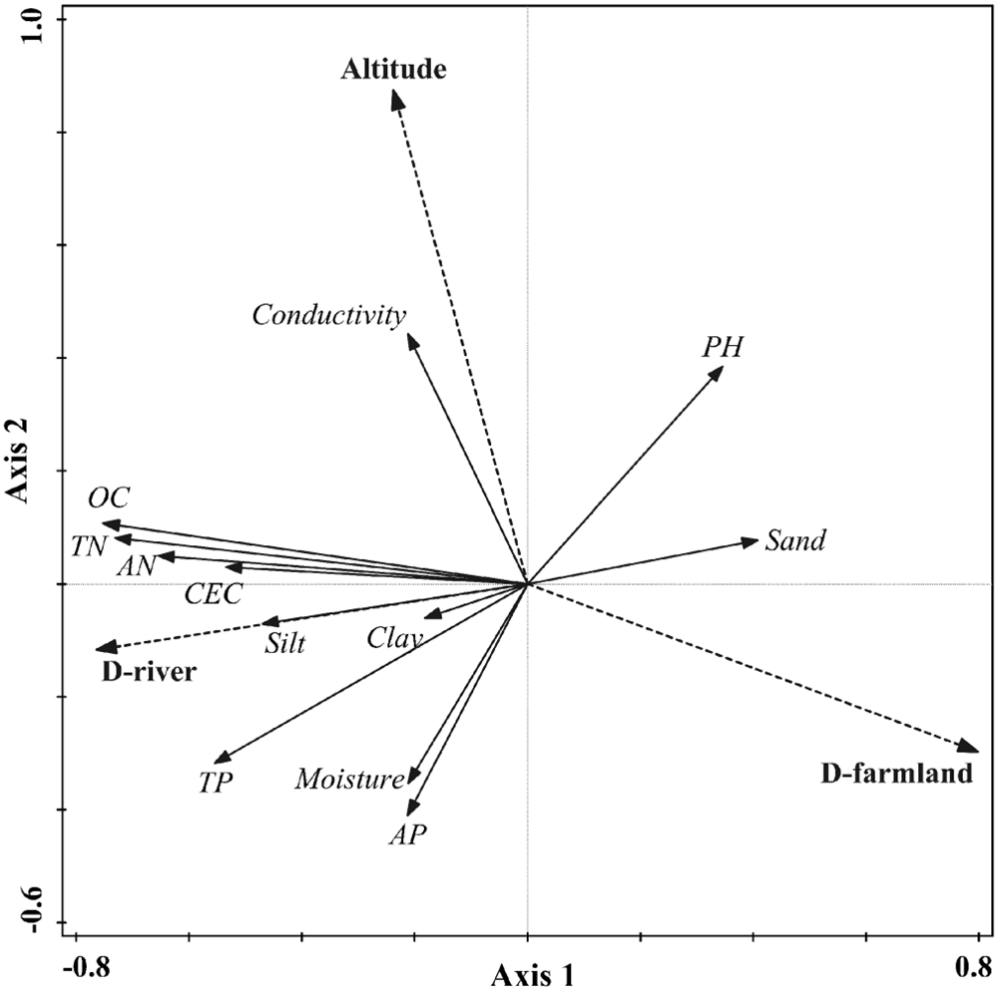
RDA results of soil physicochemical properties and environmental factors. OC: organic carbon, TN: total nitrogen, TP: total phosphorus, AN: available nitrogen, AP: available phosphorus, CEC: cation exchange capacity, D-river: distance of sampling sites from river edge, D-farmland: distance from nearest farmland.

Axis 1 and 2 of RDA were used to reflect the relationships between environmental factors and soil properties (Fig. 4). Axis 1 explained 33.69% of the total variance and axis 2 explained 4.26%, which were higher than other axes (2.33% for axis 3, and 0.35% for axis 4). Axis 1 was significantly correlated with D-river, D-farmland, and D-residence (*p* < 0.01), and axis 2 was significantly correlated with altitude (*p* < 0.01). Monte Carlo test showed that altitude (*p* = 0.014, F = 3.5), D-river (*p* = 0.002, F = 9.5), and D-farmland (*p* = 0.002, F = 13.8) were significant factors affecting soil physicochemical properties.

### Spatial autocorrelation of riparian soil nutrients

**Figure 5:**
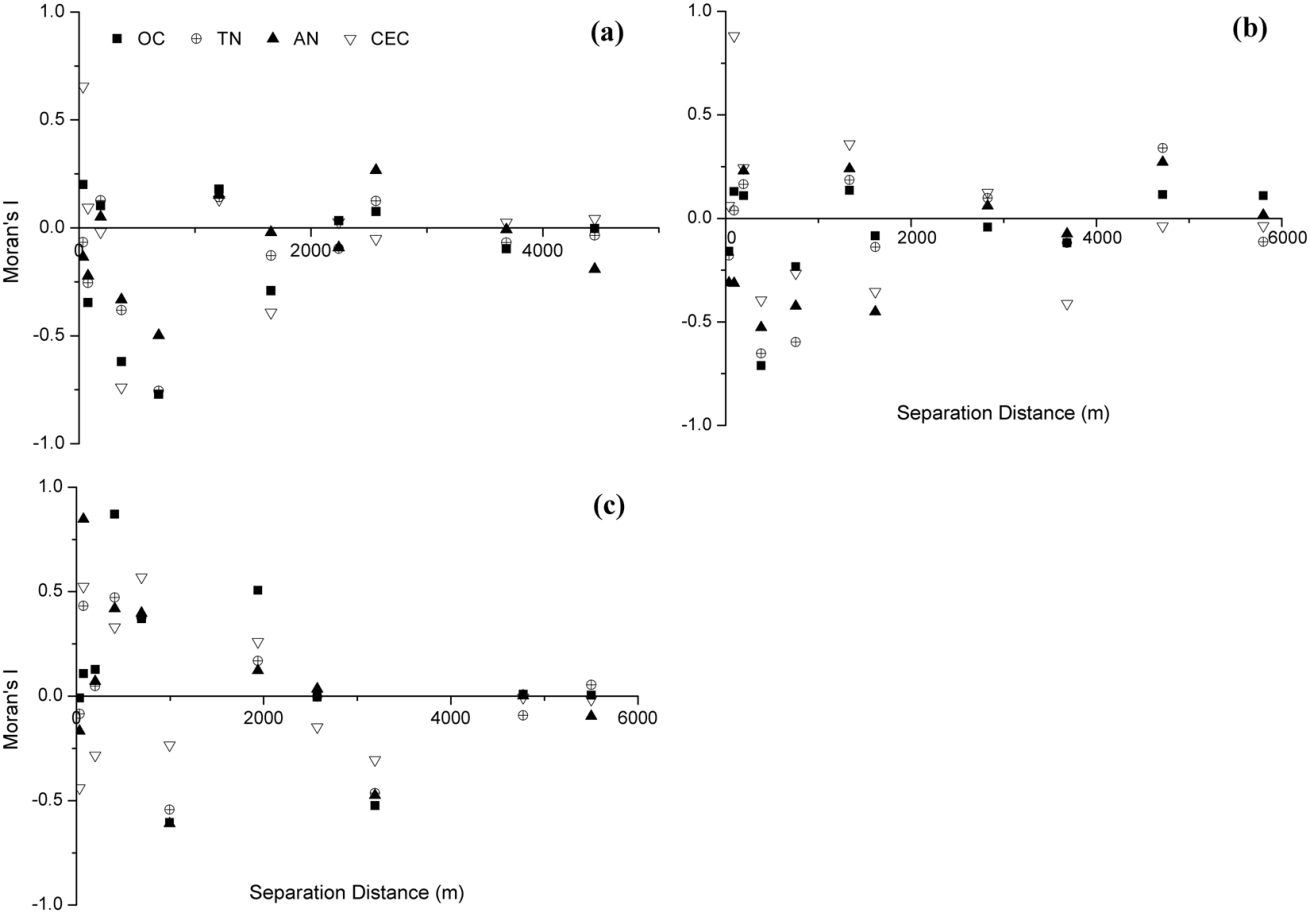
Spatial autocorrelation of riparian soil nutrients along upstream (**a**), midstream (**b**) and downstream reaches (**c**). OC: organic carbon, TN: total nitrogen, AN: available nitrogen, CEC: cation exchange capacity.

Moran’s *I* values along upstream reach decreased with increasing separation distance within the scope of 0~700 m, and the spatial autocorrelation of soil OC, TN, AN, and CEC changed from positive to negative. Moran’s *I* values approached to zero when the separation distance is greater than 1000 m, which indicated the weak spatial autocorrelation of soil nutrients (Fig. 5a).

The spatial autocorrelation of soil nutrients (except CEC) along midstream reach was not significant within the separation distance of 0~200 m. Soil nutrients separated by 300~800 m showed significant negative autocorrelation. Moran’s *I* values ranged from −0.5 to 0.5 at the separation distance greater than 1,000 m, and had no significant changed trends with the increasing distance (Fig. 5b).

Soil nutrients separated by 30~700 m mainly showed positive autocorrelation along midstream reach. At the distance of 1,000 m, soil nutrient showed significant negative autocorrelation. At the range of 2,000~3,200 m, spatial autocorrelation of soil nutrients changed from positive to negative. Soil nutrients separated by distances greater than 4,000 m showed no further autocorrelation (Fig. 5c).

According to the distribution of sampling sites, the distances among sampling sites in each sampling transect were shorter than 1,000 m, and distances among transects along each river reach were longer than 1,000 m. Therefore the autocorrelation at the separation distance shorter than 1,000 m could be used to indicate the spatial autocorrelation of soil nutrients in each sampling transect. Soil nutrients along upstream and midstream reaches mainly showed negative autocorrelation in each transect. In contrast, the spatial autocorrelation of soil nutrients was mainly positive along downstream reach.

## Discussion

The lateral heterogeneity of soil silt content, sand content, OC, CEC, TN, and AN was consistent with previous studies on natural riparian zones^14,35^, but contrary to that of riparian zones undergoing active restoration^22^. Generally, human activities in active restoration, including the use of heavy machinery, grading and site preparation activities, and the use of uniform fill materials, disturbed and homogenized soils^14^. Therefore, it seems that natural restoration in riparian zones tends to restore soil spatial heterogeneity to natural levels compared with active restoration. While active restoration is needed when dealing with heavily invaded area, where key biotic and abiotic thresholds have been crossed and resilience has been reduced^36^.

The lateral heterogeneity of riparian soil physicochemical properties displayed significant response to altitude, distance from river edge, and distance from nearest farmland. Altitude is a complex and multivariate factor that always related to substrate, flood, and vegetation^37^, which have influence on soil properties^38,39^. Along with the distance from river edge, erosion and sedimentation processes showed sharp lateral gradients^40^, resulting in the lateral heterogeneity of soil texture^19^. Soil texture, concerting with environmental factors, determined the lateral heterogeneity of soil nutrients. Previous research indicated that soils, close to river edge, with coarse material lack organic matters^41^. By contrast, areas far from the channel were characterized by high levels of fine sediment and organic matter^35^. Farmlands were the potential nutrient sources for riparian zones. Nutrients from adjacent farmlands leach or flow along the surface to stream, or enter into riparian zones and eventually accumulate in riparian soils and vegetation^41^. Nutrient leaching depend on the water-holding capacity of soils, namely, permeable soils support more intensive nutrient leaching than heavy soils^42^. Therefore, more nutrients were retained in soils with lower sand contents. On the other hand, areas close to river edge suffer from more frequent flood than that in other buffers^38^, so that nutrients in sandy soils close to river edge can be more easily flushed away.

Autocorrelation of soil physiochemical properties differed and influenced by different environmental process or factors. Soil TP and AP had no significant spatial autocorrelation, indicating a random distribution of phosphorus along lateral gradients in riparian zones. Phosphorus is an essential element for plant growth, and application of P-fertilizers is needed to overcome the deficiency of phosphorus in arable soils^43^. Spatial autocorrelation was low for soil AP in farmlands^44^. The random distribution of phosphorus indicated that fertilization history might still have impacts on soil phosphorus at the initial restoration stage after agricultural abandonment. Unlike TP and AP, soil OC, TN, AN, and CEC were spatially dependent along lateral gradients. Previous studies showed that whether fertilizers were applied or not, soil OC and TN was spatially dependent^45,46^. Anthropogenic disturbance types influence the spatial distribution of soil properties. Soil organic matter in restored riparian zones with urban land-use history was not spatially dependent, and was randomly or homogenously distributed due to the restoration activities^47^. By contrast, soil OC, TN, AN, and CEC during natural restoration with agricultural land-use history tend to distribute heterogeneously with significant autocorrelation.

Soil nutrients along upstream and midstream reaches tended to form High-Low or Low-High assemblages, while that along downstream reach tended to form High-High or Low-Low assemblages. Disturbance increases negative spatial autocorrelation in species richness and evenness^48^. The negative spatial autocorrelations of soil nutrients along upstream and midstream reaches might also be attributed to human disturbance. Along upstream and midstream reaches, farmlands within 500 m from the edges on both sides of the river were abandoned. However, riparian zones beyond 500 m were still occupied by farmlands, and external nutrient sources affected riparian soil nutrients. Therefore, human disturbance from adjacent uplands may be an inevitable factor affecting the spatial autocorrelations of riparian soil nutrients.

## Electronic supplementary material

Supplementary Information
