# Supplementary material for: Lateral heterogeneity of soil physicochemical properties in riparian zones after agricultural abandonment
Source: Sci Rep. 2018 Feb 2;8:2228. doi: 10.1038/s41598-018-20723-4 (PMC5797169; doi:10.1038/s41598-018-20723-4)
Supplement: Supplementary file 1 — Supplementary Information [file 41598_2018_20723_MOESM1_ESM.docx]

**Lateral heterogeneity of soil physicochemical properties in riparian zones after agricultural abandonment**

**Huijuan Xia^1,2,3^, Weijing Kong ^2,3*^, Xuesen Li4, Juntao Fan ^2,3^, Fen Guo^2,3^,** **Osbert Jianxin Sun^1^**

**Table S1.** Soil properties of Liaohe River Reserve. SE: standard error, OC: organic carbon, CEC: cation exchange capacity, TN: total nitrogen, TP: total phosphorus, AN: available nitrogen, AP: available phosphorus.

|  | **Range** | **Means ± SE** |
| --- | --- | --- |
| Clay (%) | 0.60 ~ 2.82 | 1.47 ± 0.07 |
| Silt (%) | 2.43 ~ 18.30 | 7.55 ± 0.46 |
| Sand (%) | 78.88 ~ 96.52 | 90.98 ± 0.52 |
| Soil moisture (%) | 1.20 ~ 29.02 | 12.44 ± 0.82 |
| pH | 6.17 ~ 8.35 | 7.46 ± 0.08 |
| Conductivity (ms.m^-1^) | 1.45 ~ 11.03 | 5.13 ± 0.26 |
| OC (mg.g^-1^) | 1.95 ~ 24.40 | 8.85 ± 0.72 |
| TN (mg.kg^-1^) | 40.39 ~ 446.21 | 190.95 ± 13.74 |
| TP (mg.kg^-1^) | 4.51 ~ 44.62 | 13.29 ± 1.19 |
| AN (mg.kg^-1^) | 2.16 ~ 43.15 | 19.44 ± 1.40 |
| AP (mg.kg^-1^) | 0.22 ~ 4.20 | 1.10 ± 0.11 |
| CEC (cmol.kg^-1^) | 4.54 ~ 139.13 | 44.74 ± 4.64 |

**Table S2.** Results of one-way ANOVA between different buffer distances. OC: organic carbon, CEC: cation exchange capacity, TN: total nitrogen, TP: total phosphorus, AN: available nitrogen, AP: available phosphorus.

| **Soil properties** | **Sum of squares** | **df** | **Mean square** | **F** | **Sig.** |
| --- | --- | --- | --- | --- | --- |
| Clay | 0.53 | 3 | 0.18 | 0.82 | 0.49 |
| Silt | 87.15 | 3 | 29.05 | 3.40 | 0.03 |
| Sand | 96.47 | 3 | 32.16 | 2.91 | 0.05 |
| Soil moisture | 186.84 | 3 | 62.28 | 1.66 | 0.19 |
| pH | 2.20 | 3 | 0.74 | 2.54 | 0.07 |
| Conductivity | 5.28 | 3 | 1.76 | 0.60 | 0.62 |
| OC | 144.28 | 3 | 48.09 | 7.33 | 0.00 |
| TN | 197236.57 | 3 | 65745.52 | 10.38 | 0.00 |
| TP | 595.63 | 3 | 198.55 | 2.42 | 0.08 |
| AP | 8.37 | 3 | 2.79 | 3.87 | 0.02 |
| AN | 1984.55 | 3 | 661.52 | 11.28 | 0.00 |
| CEC | 17187.92 | 3 | 5729.31 | 6.75 | 0.00 |
